# Presentation of retinoblastoma in pregnancy

**DOI:** 10.4103/0301-4738.62649

**Published:** 2010

**Authors:** Varsha S Nandedkar, Anil R Joshi, Namrata Kabra, Neha N Deshpande

**Affiliations:** Department of Ophthalmology, Government Medical college, Aurangabad, Maharashtra, India

**Keywords:** Chemoreduction, enucleation, flexner-wintersteiner rosettes, homer-wright rosettes, immunohistochemistry, retinoblastoma

## Abstract

A 22-year-old female in her third trimester of pregnancy was referred to our department for sudden loss of vision with a painful blind eye. It was diagnosed as retinoblastoma clinically and radiologically. Histopathology and immunohistochemistry confirmed the diagnosis. This case is one of its kind because retinoblastoma occurring during pregnancy had not been reported in literature so far.

Retinoblastoma is the most common intraocular tumor in childhood; occurring before the age of two years and rarely after the age of four years. Literature review shows only 23 cases in adults[1] [Table 1]. However, not a single case of retinoblastoma in pregnancy has been reported.

**Table 1:** Cases of retinoblastoma in adults

Author	Age (Yr)	Duration of S/S (Mnth)	Sex	Tumor Size	Tumor location	Rosettes	Retinocyte
Maghy	20	12	F	Whole eye	Whole Eye	FW	NS
Verhoeff	48	6	M	10×15mm	Superonasal	FW	NS
McCrea	20	NS	M	NS	NS	Yes but NS	NS
Rasmussen	48	4	M	16×14mm	Anterior to equator	No	NS
O'Day	29	NS	M	NS	Anterior to equator	NS	NS
Rychener	38	54	F	6.5×7mm	Posterior pole	FW	NS
Arseni *et al*.	53	4	M	NS	Posterior pole	FW	NS
Mehera *et al*.	45	24	M	NS	Anterior to equator	FW	NS
Finlay *et al*.	74	6	F	NS	NS	Yes but NS	NS
Ohara *et al*.	43	48	F	3.8×3.8	Optic nerve head	HW	NS
Makley	52	60	M	20×25mm	Whole Eye	FW	NS
Lash	40	60	F	NS	NS	No	Yes
Perz *et al*.	56	5	M	NS	NS	Yes but NS	NS
Kremlicka *et al*.	42	4	M	8×18mm	Anterior to equator	FW	NS
Berkeley *et al*.	60	3	M	Whole eye	Whole Eye	No	NS
Takahashi *et al*.	26	6	F	NS	Superior	HW	NS
Neronova-Kotova	46	24	F	NS	NS	Yes but NS	NS
Nork *et al*.	29	NS	F	8×10mm	Nasal	Yes but NS	Yes
Mietz *et al*.	26	3	F	12×2mm	Anterior to equator	HW	NS
Biswas *et al*.	32	24	M	10×8mm	Posterior pole	HW	NS
Biswas *et al*.	21	1	M	12.5×11.5mm	Superotemporal	NS	NS
Biswas *et al*.	25	1	F	12.5 ×11.5mm	Superonasal	No	NS
Alexander *et al*.	21	2	F	16 ×16mm	Inferotemporal	FW	Yes

NS- Not Specified, FW- Flexner –Wintersteiner Rosetts, HW- Homer - Wright

## Case Report

**Figure 1:**
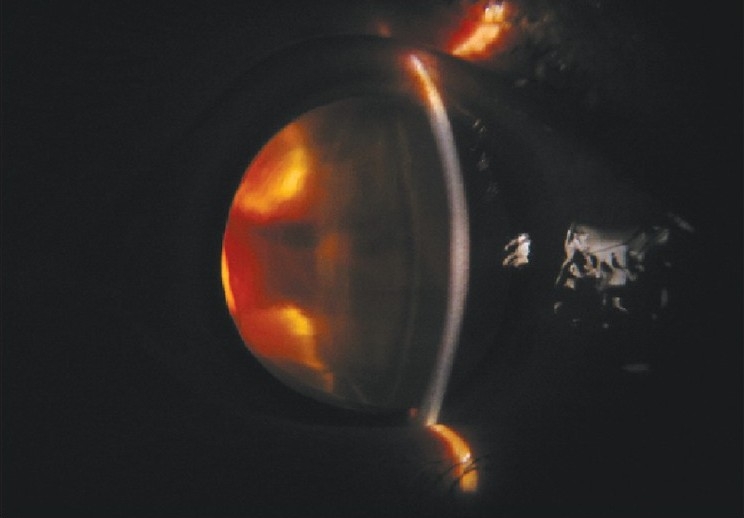
On slit-lamp examination findings show circumcorneal congestion, shallow anterior chamber with aqueous cells and dilated pupils

**Figure 2:**
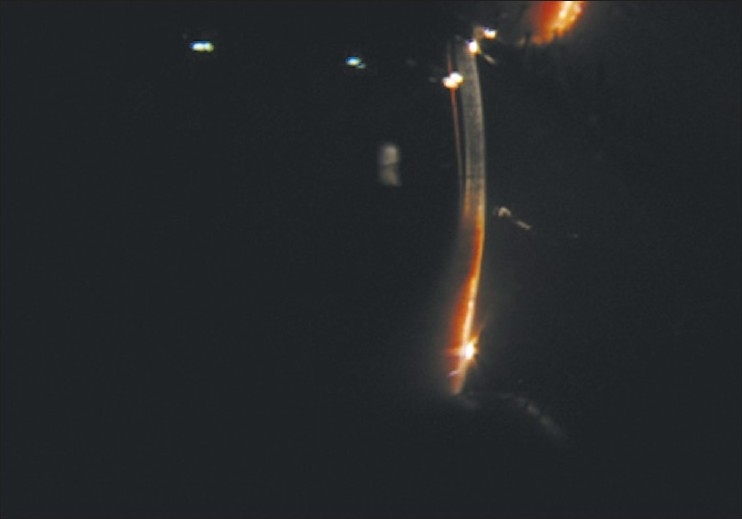
Slit lamp view of anterior chamber for keratic precipitate, aqueous cells and flare

**Figure 3:**
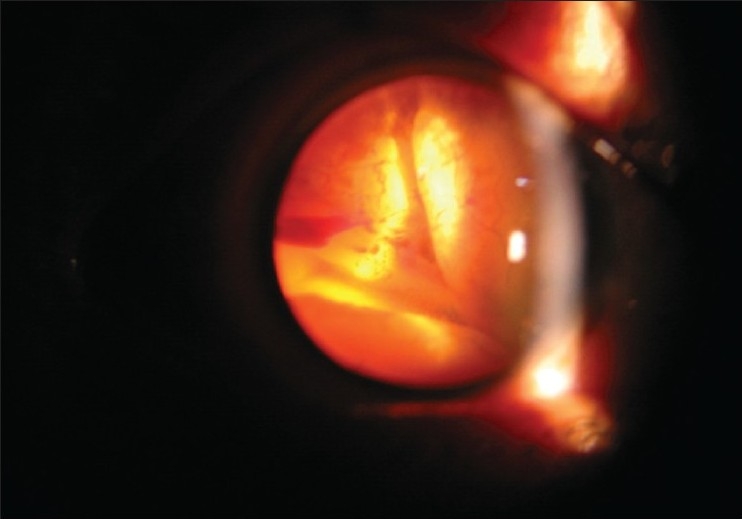
On slit lamp bi-microscopic examination vitreous hemorrhage, exudative retinal detachment with intra-retinal hemorrhages were found

A 22-year-old female in third trimester of pregnancy was referred to our department from the obstetrics ward for sudden loss of vision in right eye. Her visual acuity was ‘no perception of light’. She had no history of trauma, surgery or use of spectacles. On the slit-lamp examination, findings were circumcorneal congestion, keratic precipitates, shallow anterior chamber with aqueous cells [Figs. 1 and 2], and dilated fixed pupil. Intraocular tension was 28mm of Hg. On indirect ophthalmoscopic examination, vitreous hemorrhage and exudative retinal detachment with intra-retinal hemorrhages were found [Fig. 3]. Patient was advised fine needle aspiration biopsy but lost on follow-up and turned up three months after delivery complaining of pain, redness of right eye with headache associated with ciliary staphyloma.

**Figure 4:**
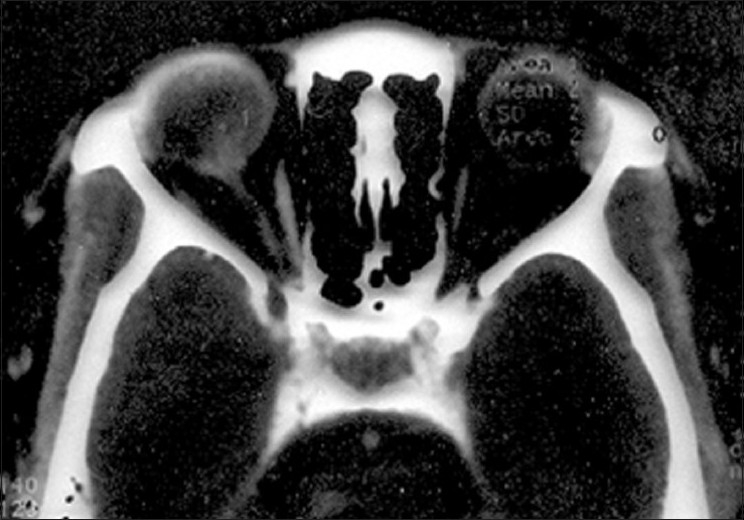
On contrast enhanced computed tomography (CECT) of orbit, a contrast enhanced mass at posterior pole with absence of calcification was reported

**Figure 5:**
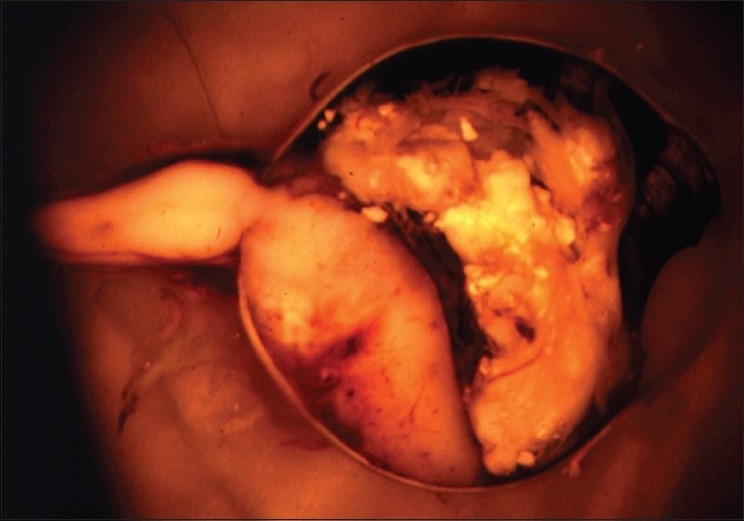
Gross specimen shows whole of the posterior segment involvement by tumor with infiltration of the optic nerve

On contrast enhanced computed tomography (CECT) of orbit, a contrast enhanced mass at posterior pole with absence of calcification was reported [Fig. 4]. The patient underwent enucleation. Gross specimen showed whole of the posterior segment involvement by the tumor with infiltration of the optic nerve [Fig. 5].

**Figure 6:**
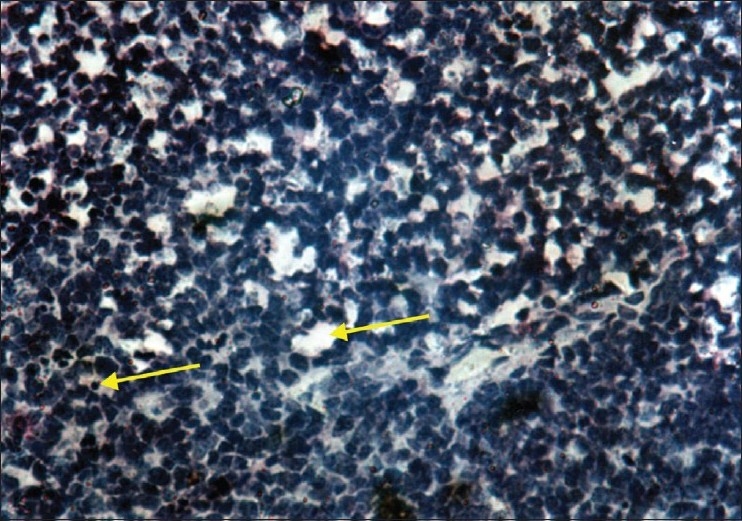
This histopathological slide, stained with Hematoxilline and eosine seen under 100× magnification shows occasional rosettes in tumor tissue. Rosettes are shown by arrow

**Figure 7:**
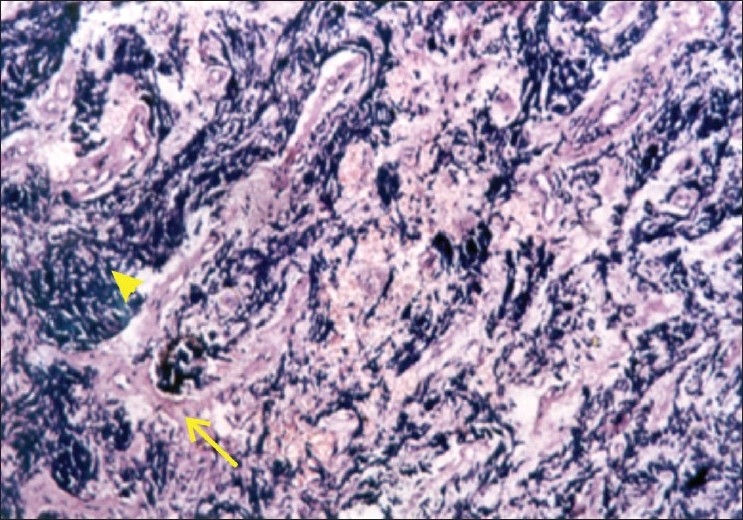
This is histopathological slide, stained with H & E seen under 40× magnificationshowing eosinophilic optic nerve tissue (shown by arrow) infiltration by basophilic tumor cells (shown by arroe-head).

Histopathological examination showed small round cells with hyperchromatic nuclei arranged in cords along with undifferentiated cells. Occasional rosettes and areas of necrosis were found [Fig. 6]. Calcification was not seen. Ciliary body, choroid and sclera showed infiltration. Optic nerve was infiltrated [Fig. 7] up to the cut section. With these histopathological findings, diagnosis of small cell tumor was made. There was no evidence of mature retinocytoma cells. Immuno-histochemistry was performed for exact diagnosis which was positive for neuron specific enolase and negative for cytokeratin, vimentine, S-100. Patient was classified as Group E retinoblastoma according to International Classification of Retinoblastoma.

She received chemotherapy with carboplatin, vincristin, etoposide for six months and radiotherapy of 200cGy for four weeks to orbit. Although this was a case of non-hereditary retinoblastoma (due to age), contralateral eye of the patient, her first degree relatives and her newborn child were checked for ocular disease and found normal. Patient was followed for one year. By the end of one year CECT-orbit showed infiltration up to the orbital apex. She refused further treatment and died after four years probably due to metastatic complications, exact details are not available.

## Discussion

Retinoblastoma is a rare tumor in adults. Literature review shows only 23 cases of retinoblastoma above 20 years.[1] [Table 1] Retinoblastoma during pregnancy had not been reported.

On literature review through journals, cross references from cases and Pubmed search, we came to the some conclusions:

Age of presentation of retinoblastoma in adults was 20-74 years. Oldest patient was a 74-year-old female reported by Finlay [Table 1]. Most common age at presentation was 20-30 years (nine cases), followed by 41-50 years (six cases). Mean age was 39 years. No significant difference was found in male (12 cases) and female (11 cases) [Table 1].

No case was reported in the pregnancy although Takahashi *et al*. reported one case which was presented six months post-partum.[2] This 26 year old female had acute presentation with photopsia, diminution of vision and field defect.

Clinical presentation is also different in adults as majority of them presented with diminution or loss of vision. Flashes, floaters, photopsia were also presenting features. Very few presented with leucocoria or squint which are commoner presentations in children. On indirect ophthalmoscopy, retinal detachment was the most common finding followed by vitreous hemorrhage, which may be non-resolving.[3]

Majority of the tumors were differentiated showing rosettes (17 cases). Completely undifferentiated tumors needing immunohistochemistry were few (four cases). Detailed histopathological findings have not been mentioned in two cases. Though calcification is supposed to be one of the criteria for diagnosis of retinoblastoma, it was never seen in adult cases including our case.

Retinocytoma is a benign tumor of retina. It has got the same genetic characteristics and hereditary pattern as retinoblastoma. Singh and colleague had stated that malignant transformation rate of retinocytoma is four per cent.[4] It is unknown if retinoblastoma in adults arises as malignant transformation of retinocytoma. Only two cases in literature documented well demarcated areas of retinocytoma. In our case no such areas of retinocytoma were found.

In a normal person, circulating mutagens are known to bind to cell and deactivate the retinoblastoma protein. If the patient has only one working copy, deactivation of this gene may affect cellular signaling and results in retinoblastoma. In pregnancy, the patient may have excessive circulating mutagens which may be responsible for retinoblastoma.[5] Immunosuppression in pregnancy may be responsible for fast growth and dissemination of the tumor. Treatment of malignancy in pregnancy is difficult. Not much information is available in the literature regarding this. Vision preserving techniques like cryotherapy, transpupillary thermotherapy and laser can be used safely. Enucleation under local anesthesia can also be done. External beam radiation is contraindicated. If single agent chemotherapy is given during the first trimester of pregnancy, incidence of teratogenic effect is 10%. It increases to 25% when multiple chemotherapeutic drugs are used. In the 2^nd^ and 3^rd^ trimester, even though there are less chances of teratogenic effect, low birth weight, intra-uterine growth retardation, intra-uterine death and post-natal problems are common.[6] Chemotherapeutic agents such as carboplatin, vincristin, etoposide can be used with such risks mentioned above.

Diagnostic dilemma often exists as retinoblastoma is not expected in adults. In such a situation, differential diagnoses are- amelanotic melanoma, metastatic carcinoma, lymphoma, leukemia, endophthalmitis, panophthalmitis and inflammatory diseases of retina.[7] Diagnosis of retinoblastoma is difficult when inflammation, vitreous hemorrhage and cataract are the presenting features.[8]

In developed countries, five-year disease-free survival rate is 90-95%. These survivors have substantially increased risk of death from non-retinoblastoma malignancy pinealoblastoma, tri-lateral retinoblastoma, bony and soft tissue sarcomas. This risk is 20% in 25 years.

Perhaps this is the only case of adult retinoblastoma presenting during pregnancy in literature. If in future, more cases are presented this way, it would highlight the relationship between pregnancy and retinoblastoma.
